# Validated Matrix Matched Quantification of Ethyl Chloride in Postmortem Biological Samples Using HS-GC-FID: Lung as the Optimal Tissue and Temporal Detection Dynamics

**DOI:** 10.3390/toxics13121024

**Published:** 2025-11-27

**Authors:** Halit Canberk Aydogan, Ali Rıza Tümer, Ramazan Akçan, Mahmut Şerif Yıldırım, Mukaddes Gürler

**Affiliations:** 1 Department of Forensic Medicine, Ordu University Training and Research Hospital, Ordu 52200, Türkiye; 2 Department of Forensic Medicine, Faculty of Medicine, Hacettepe University, Ankara 06100, Türkiye; tumeralir@hotmail.com (A.R.T.); akcanmd@hotmail.com (R.A.); 3 Department of Forensic Medicine, Faculty of Medicine, Uşak University, Uşak 64100, Türkiye; dr.msyildirim@gmail.com; 4 Departments of Forensic Medicine and Medical Biochemistry, Faculty of Medicine, Hacettepe University, Ankara 06100, Türkiye; dr.mkdds@gurler.eu

**Keywords:** ethyl chloride, inhalant abuse, ice spray, forensic toxicology, forensic medicine

## Abstract

Ethyl chloride, a volatile anesthetic with high abuse potential, remains forensically undercharacterized postmortem. In an inhalation model (*n* = 30), male Wistar rats were exposed to 86,000 ppm ethyl chloride under real-time PID monitoring; blood, lung, liver and brain (plus exploratory adipose, kidney, muscle) were sampled at 0, 2, 4, 6 and 12 h postmortem. A matrix-matched HS-GC-FID method was validated (Eurochem): linearity (R^2^ = 0.9947–0.9965), LOD 0.01–0.02 ng/μL, LOQ 0.04–0.06 ng/μL, precision RSD 3.9–5.1%, recovery 90–104%, full selectivity against common volatiles. Lung yielded the highest concentrations overall; a significant decline occurred in lung between 2 h and 4 h (Pillai’s Trace *p* = 0.034). Concentrations became increasingly irregular ≥6 h across tissues. Early autopsy sampling, preferably within ≤6 h, optimizes ethyl chloride detectability. The validated matrix-matched HS-GC-FID protocol provides a cost-effective, robust alternative to MS platforms for volatile screening in routine forensic practice and supports prioritizing lung for analysis.

## 1. Introduction

In recent years, volatile substance abuse has emerged as a significant and chronic public health issue worldwide, with substantial social and medical implications [1,2]. Among these substances, ethyl chloride (chloroethane, CH_3_CH_2_Cl) a volatile, flammable anesthetic with a sweet, ether-like odor, has gained increasing forensic relevance due to its rising misuse potential. Historically utilized as a general anesthetic, ethyl chloride is now commonly used as a local anesthetic across various medical fields, including dentistry, orthopedics, sports medicine, and dermatology, primarily in the form of an ice spray. Due to its high anesthetic potency and low boiling point (12.3 °C), ethyl chloride produces rapid local cooling upon application, resulting in effective transient analgesia [3,4,5]. Available commercially as a cooling pressure spray, its accessibility and affordability contribute to its potential for abuse. Abuse typically involves direct inhalation, either nasally or orally, often by releasing the gas into a bag [6].

Given its affordability, accessibility, and rapid psychoactive effects, ethyl chloride has become increasingly susceptible to misuse, particularly among adolescents. In response to growing public health concerns, some countries, such as the United Arab Emirates (UAE), have imposed regulatory restrictions on retail distribution and initiated awareness campaigns aimed at curbing inhalant abuse [7]. These developments underscore the urgent need for forensic and toxicological frameworks capable of addressing ethyl chloride-related fatalities. Moreover, recent trends highlight an alarming increase in inhalant abuse, particularly among adolescents, driven by socio-economic inequalities and insufficient support systems [8]. Vulnerable populations, such as street-involved youth, are disproportionately exposed to exploitative environments that amplify substance use behaviors. Evidence suggests these behaviors stem not solely from individual or familial shortcomings but are deeply entrenched in systemic disparities, including poverty, limited access to education, and inadequate healthcare infrastructure [9]. In addition to socio-environmental challenges, the neurotoxic and systemic health impacts of inhalant abuse further compound the public health burden. These combined effects necessitate urgent action to address both the root causes and systemic consequences of such behaviors. Taken together, such findings emphasize the necessity of reframing inhalant-use behaviors through a socio-environmental perspective, challenging reductionist narratives that attribute such actions merely to deviance or pathology.

The pharmacokinetics of ethyl chloride reveal its high lipophilicity, allowing it to quickly cross the blood–brain barrier, leading to central nervous system (CNS) depression and a transient euphoric effect. However, this rapid absorption and high lipid solubility also results in significant adverse effects. Acute exposure can cause cardiac arrhythmias by increasing endogenous epinephrine levels and can lead to severe neurological and psychological manifestations such as confusion, hallucinations, and ataxia. Chronic exposure has been linked to profound cognitive deficits, including memory impairment and hepatic dysfunction, contributing to severe neurotoxicity and systemic health issues [10]. Despite its widespread use and abuse potential, the lethal concentration of ethyl chloride in humans remains undetermined [11]. Although its clinical and toxicological profiles have been partially characterized, critical forensic parameters such as the lethal concentration and postmortem detectability remain underexplored. Literature on fatalities associated with ethyl chloride inhalation abuse is sparse, with only three reported cases of death [12,13,14]. Given the multifaceted challenges associated with the systemic impacts and toxicological complexities of ethyl chloride, addressing these issues requires methodological innovations and interdisciplinary approaches. A recent review highlighted the inadequacy of traditional toxicological methods in identifying volatile substances like ethyl chloride in postmortem settings, emphasizing the need for advanced detection methodologies [15].

Due to its high volatility and rapid redistribution postmortem, ethyl chloride poses significant challenges in forensic toxicology. Existing case reports lack systematic evaluation of its behavior after death, particularly regarding which tissues retain the compound and for how long. Despite its pervasive misuse and documented association with ethyl chloride-related fatalities, there remains a critical paucity of experimental research addressing the postmortem behavior of ethyl chloride. Previous studies primarily have focused on the acute toxicological effects of ethyl chloride rather than its postmortem dynamics, leaving a significant gap in understanding its forensic relevance. While case reports documenting human fatalities linked to ethyl chloride misuse exist, ethical and toxicological constraints have precluded experimental studies on humans, further underscoring the necessity for robust animal models to bridge this knowledge gap. Specifically, the literature lacks comprehensive data on which biological samples are most suitable for toxicological analysis and the optimal time intervals postmortem during which ethyl chloride can be reliably detected. This knowledge gap significantly hampers forensic toxicology practices, particularly in cases where volatile substances play a central role in determining the cause of death.

This study aims to address critical gaps in forensic toxicology by investigating the postmortem distribution and temporal persistence of ethyl chloride across multiple biological matrices. Specifically, it seeks to identify the most reliable tissues for postmortem toxicological analysis, determine the optimal sampling intervals, and develop a rigorously validated analytical framework using headspace gas chromatography with flame ionization detection (HS-GC-FID). To achieve these objectives, an experimental rat model was established to simulate fatalities resulting from the inhalation of ethyl chloride via ice spray. Biological samples, including blood and various organs, were systematically collected at predefined postmortem intervals to evaluate ethyl chloride levels. The study employed matrix-matched calibration and cold-chain protocols to ensure methodological reliability and analytical precision. The use of HS-GC-FID represents a significant methodological advancement, offering high sensitivity, specificity, and reproducibility in the quantification of volatile compounds in forensic contexts. By elucidating the temporal dynamics of ethyl chloride detectability, the findings contribute valuable empirical data to refine postmortem toxicological protocols. To the best of our knowledge, no prior study has experimentally validated a matrix-matched HS-GC-FID protocol for postmortem ethyl chloride detection under controlled exposure conditions.

Ultimately, this study enhances the interpretative capacity of forensic investigations involving volatile substance abuse and supports the development of standardized sampling strategies. By bridging key methodological gaps, it lays a robust foundation for improved medicolegal practices and strengthens the integration of experimental data into practical forensic toxicology.

## 2. Materials and Methods

### 2.1. Chemicals

Ethyl chloride 1000 µg/mL (Sigma Aldrich^®^ St. Louis, MO, USA, standard substance), n-propanol (Sigma Aldrich^®^ St. Louis, MO, USA, internal standard), PIC^®^ ice spray (PIKDARE S.p.A., Gazzada Schianno, Varese, Italy), distilled water (MES Mp Minipure İzmir, Turkey), and dry ice were utilized in this study. 

### 2.2. Animal Experiment and Ethyl Chloride Ice Spray Administration

The study employed 400 mL PIC^®^ ice spray cans, which utilize a dual-phase aerosol system. The active liquid phase comprises 89% ethyl chloride, providing the primary anesthetic and cooling effects. The propellant phase, accounting for 95.88% of the total aerosol composition, includes a butane/propane mixture pressurized at 3.2 bar, along with 0.06% menthol, 2.06% isopropyl alcohol, and 2% isopropyl myristate.

This ice spray system ensures efficient pressurization, atomization, and delivery of the active and auxiliary components in aerosol form. Male Wistar Albino rats (10–14 weeks old, 250–300 g, n = 10) were housed in a 120 L Microtest^®^ MIT 120 chamber (Microtest, Ankara, Turkey), which was maintained at an ambient temperature of 23 °C ± 1 °C and a relative humidity of 40% ± 5%. These environmental conditions were consistently regulated throughout the experiment to ensure controlled exposure and reproducibility. Animals were housed under controlled environmental conditions (23 ± 1 °C; 40 ± 5% RH) with standard husbandry. No procedures causing pain or distress were performed beyond the exposure protocol; euthanasia was not required as exposure was lethal under LC_50_-based modeling. 

**Figure 1:**
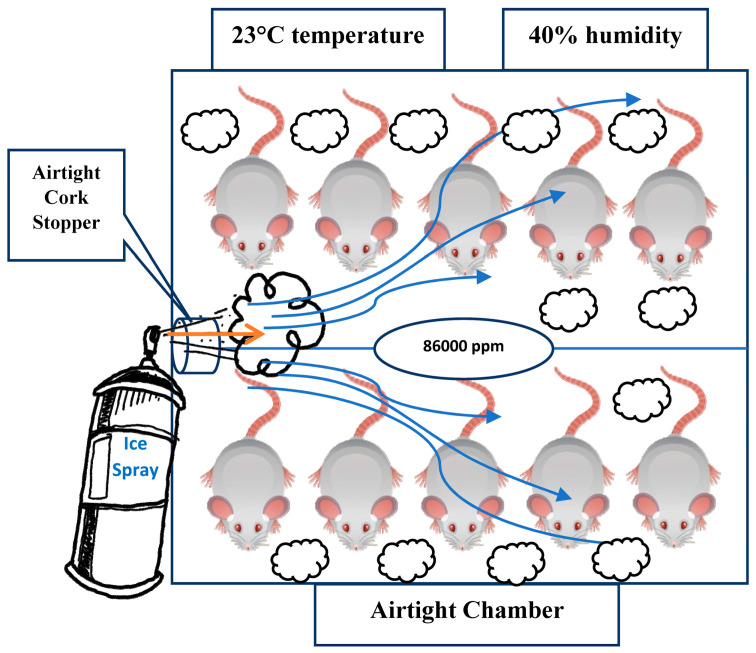
Experimental Setup for Ethyl Chloride Inhalation in Rats.

The chamber featured a round-shaped lid on the right side, which was replaced with an airtight cork stopper to facilitate gas administration. The ambient temperature inside the inhalation chamber was consistently maintained at 23 °C and monitored throughout the experimental procedure using an integrated digital thermometer, ensuring stable and controlled experimental conditions. A schematic representation of the ethyl chloride inhalation setup for the rats in the chamber is provided in Figure 1.

Following OECD testing guidelines and considering the LC_50_ value of 85,747 ppm for rats, a concentration of 86,000 ppm of ethyl chloride was administered as an inhalant. This dosage was determined to ensure lethality while taking into account the rats’ anatomical and metabolic variability [16,17].

To ensure precise and consistent exposure throughout the inhalation period, a real-time gas concentration monitoring system was employed using a photoionization detector (PID) (MiniRAE 3000, RAE Systems, San Jose, CA, USA). The device operates based on ultraviolet ionization of volatile organic compounds (VOCs) and was precalibrated with isobutylene (100 ppm standard gas) in accordance with the manufacturer’s protocol. A correction factor (CF) of 0.9 was applied for ethyl chloride, as per the RAE Systems calibration database. The PID continuously measured the VOC concentration within the chamber at 10 s intervals, and the data were recorded and exported using ProRAE Studio II (Version 1.9) software. During all exposure sessions, the recorded values demonstrated a stable ethyl chloride concentration averaging 86,000 ppm (±3.2%), thereby verifying the achievement of the predefined target concentration aligned with LC_50_-based toxicological thresholds for rodents.

The application of this real-time detection protocol effectively eliminated uncertainties associated with theoretical dosing calculations and ensured homogeneous and reproducible gas distribution across all test subjects. This methodological enhancement aligns with internationally accepted inhalation toxicology standards and exposure control strategies and significantly strengthens the internal validity and reproducibility of the experimental design [18,19,20].

To optimize gas dispersion and prevent fluctuations, the chamber was maintained at a constant temperature and humidity to minimize volatilization inconsistencies, sealed with an airtight cork stopper to prevent unintended diffusion, and actuated in a controlled manner, ensuring homogeneous exposure to all subjects within the chamber.

The airtight glass at the front of the chamber allowed for detailed observation and recording of the rats’ death times. The procedure was conducted in three separate trials to ensure reproducibility. The results indicated that the rats succumbed to the ethyl chloride within an average of 24 min and 20 s (SD = ±2.4 min), with no significant variation in death times.

Postmortem blood and tissue samples were collected from the rats at five time points: immediately (0 h), and at 2, 4, 6, and 12 h postmortem. Autopsies were performed on six rats at each of these time intervals, resulting in a total of 30 rats being studied. The selection of specific postmortem intervals was guided by the well-documented difficulty of detecting inhalant toxicological substances beyond 12 to 24 h after death. Ethyl chloride, being both highly volatile and lipophilic, is particularly susceptible to rapid postmortem redistribution and degradation, necessitating precise sampling within the early postmortem period to ensure accurate toxicological analysis [21]. Additionally, the U.S. Environmental Protection Agency (EPA) provides Health Effects Test Guidelines for acute inhalation toxicity, detailing procedures to determine the adverse effects following a single, uninterrupted exposure by inhalation over a short period (24 h or less) [22]. Furthermore, the selection of 0, 2, 4, and 6 h intervals in this study aligns with Keskin’s study, who employed a similar timeframe in postmortem investigations of volatile substances, reinforcing the appropriateness of these intervals for the detection and analysis of ethyl chloride [23].

All experimental procedures were reviewed and approved by the institutional animal ethics committee and were conducted in full compliance with national and international ethical standards.

### 2.3. Samples

All experimental animals were promptly transferred from the exposure chamber to the necropsy area, and systematic autopsies were performed at the designated postmortem time points. Samples were collected in a standardized manner from the following anatomical regions: cardiac blood (left ventricle), lung tissue (left lobe), liver tissue (right lateral lobe), perirenal adipose tissue (adjacent to the left kidney), left kidney, muscle tissue (left biceps femoris), and brain tissue (left cerebral hemisphere). The entire dissection and sampling process for each rat was completed within 5 min to minimize potential postmortem redistribution and analyte loss. Each biological sample, comprising 225 µL of blood or 225 mg of solid tissue, was immediately transferred into 10 mL HS-GC-FID vials. Subsequently, 25 µL of internal standard solution (n-propanol, 1:100 dilution) was added to each vial.

To minimize any external exposure to volatile interferents, all necropsies were performed in a dedicated autopsy room where the use of alcohol-based disinfectants, organic solvents, and aerosolized sprays was strictly avoided during sampling. Stainless-steel instruments and glassware were rinsed with distilled water only, and no ethanol, methanol, acetone, or isopropanol was used in the immediate sampling area. The rapid transfer of blood and tissues into sealed HS-GC vials under cold-chain conditions (dry ice) was intended to reduce evaporative loss of ethyl chloride and other volatiles during handling. The absence of unexpected peaks or co-eluting signals in blank control samples further supported that there was no measurable environmental contamination by common volatile interferents. The internal standard was pre-diluted in deionized water solely to ensure pipetting homogeneity and precision during microvolume transfer. This step did not influence the quantitative reliability of the results, as all analytical measurements were performed using calibration curves constructed in matrix-matched biological environments. To ensure analytical rigor and account for potential matrix effects, method validation was conducted using blank (non-exposed) rat tissues from four key biological compartments blood, lung, liver, and brain. These compartments were selected based on their physiological relevance to the absorption, metabolism, and retention of volatile substances. Blank tissues were obtained from control rats euthanized under identical ethical and environmental conditions and were spiked with known concentrations of ethyl chloride under cold-chain conditions to prevent analyte volatilization. The same HS-GC-FID protocol was applied to both calibration and experimental samples, ensuring analytical consistency. Although additional tissues adipose, kidney, and muscle were collected and analyzed for exploratory purposes, matrix-matched calibration was not feasible for these due to the limited availability of sufficient high-purity blank tissues and the heightened risk of baseline volatility interference. Consequently, while quantitative measurements in these matrices were interpreted with caution, their inclusion provided valuable comparative insights into the distributional behavior and toxicological relevance of ethyl chloride in diverse postmortem compartments. The use of an animal model, rather than human autopsy tissues, enabled tightly controlled exposure parameters and standardized sampling intervals, ensuring experimental reproducibility and eliminating confounding variables inherent to real-world forensic cases. While interspecies differences must be acknowledged, the present design allows for systematic characterization of ethyl chloride’s distribution and temporal behavior, serving as a robust experimental foundation for future translational and validation studies.

### 2.4. Instrumental Analysis

Samples were analyzed using HS-GC-FID. Throughout the analytical process, the calibration and validation of the HS-GC-FID system were rigorously performed. Minor limitations, such as temperature control limitations and sensitivity to high-boiling compounds, were identified; however, these were effectively mitigated through method optimization and routine maintenance, thereby ensuring robust and reproducible results. The instrumental parameters outlined in Table 1 were meticulously optimized to achieve reliable chromatographic separation and quantification of ethyl chloride, as substantiated by matrix-matched calibration procedures conducted under validated analytical conditions. Table 1 provides comprehensive details regarding the autosampler, inlet, column and detector settings, which were critical for the accurate quantification of ethyl chloride across various postmortem biological matrices.

### 2.5. Preparation of Standard Working Solutions

To ensure accurate and reproducible calibration standards, crucial for the reliable quantification of ethyl chloride in subsequent analyses, preparation of standard working solutions was as follows. To address the volatility and low boiling point of ethyl chloride, dry ice was employed to maintain a cold environment throughout the preparation process. Dry ice, the solid form of carbon dioxide, has a temperature of −78.5 °C, effectively preventing the evaporation of ethyl chloride during handling and preparation [24].

A 1000 µg/mL ethyl chloride reference standard solution was prepared using a methanol mixture at a 1:5 ratio (*v*/*v*). The internal standard, n-propanol with a purity of 99.8%, was diluted in distilled water to achieve a 1/100 concentration. For calibration, five different concentration levels of ethyl chloride were prepared, ranging from 0.2 to 2 ng/µL. Calibration standards were prepared by spiking blank biological matrices (blood, lung, liver and brain) with ethyl chloride and internal standard under cold-chain conditions to prevent volatilization. All calibration and quality-control vials were immediately sealed with crimp caps and kept on dry ice until analysis to further limit natural evaporation of ethyl chloride during preparation and handling.

### 2.6. Method Validation

A matrix-matched method validation protocol was developed in accordance with the Eurochem Guide to evaluate the performance characteristics of ethyl chloride quantification in postmortem biological tissues [25]. Four matrices blood, lung, liver, and brain were selected based on their relevance in forensic toxicology and their differing biochemical properties. Matrix-matched validation was specifically performed using blank blood, lung, liver, and brain tissues to address the matrix effects arising from endogenous volatiles and non-volatiles in real forensic samples.

Calibration standards were prepared by spiking blank biological matrices with ethyl chloride at five concentration levels: 0.2, 0.5, 1.0, 1.5, and 2.0 ng/µL. Each concentration level was analyzed in triplicate using an HS-GC-FID system. The internal standard (n-propanol, 25 µL at 1:100 dilution) was added to all vials to control for injection variability and instrumental drift. Calibration curves were generated for each matrix using least-squares linear regression.

Method precision and accuracy were assessed by analyzing quality control (QC) samples at three concentration levels (0.2, 0.8, and 2.0 ng/µL), with five replicates per level analyzed intraday and interday across three separate days. Precision was expressed as relative standard deviation (RSD%), and accuracy was calculated as percent deviation from nominal concentrations. Acceptance criteria were set at ≤±20%, in accordance with the Eurochem Guide.

Limits of detection (LOD) and limits of quantification (LOQ) were determined based on replicate (n = 10) analysis of low concentration spiked samples. LOD and LOQ were defined as the concentrations corresponding to signal-to-noise ratios of 3 and 10, respectively.

Selectivity was evaluated by analyzing each blank matrix spiked with ethyl chloride in the presence of common volatile interferents, including ethanol, methanol, acetone, and isopropanol. Baseline separation of all compounds both in standard solution and fortified biological matrices was confirmed by chromatographic analysis. All biological samples and calibration solutions were stored in sealed 10 mL HS-GC vials at −20 °C and transported on dry ice between the necropsy room and the analytical laboratory. Under these conditions, no systematic drift in ethyl chloride peak area ratios was observed across analytical batches, indicating that trace amounts of ethyl chloride remained stable during the short-term storage period relevant to this study. Absolute recovery was assessed by comparing the peak areas of spiked matrix samples to the corresponding theoretical concentrations.

All sample preparations were conducted under cold-chain conditions using dry ice, and analyzed under optimized conditions using HS-GC-FID. Due to the limited availability of clean blank adipose, kidney, and muscle tissues from non-exposed animals, matrix validation was focused on the four most diagnostically relevant matrices: blood, lung, liver, and brain. These matrices are also the primary sites of distribution and metabolism for volatile substances.

### 2.7. Statistical Analysis

Statistical analyses were conducted to evaluate the temporal variation and tissue-specific distribution of ethyl chloride concentrations across four biological matrices. All statistical procedures were performed using IBM SPSS Statistics Version 23 and the MVN package in R [26]. Univariate normality for each matrix (blood, lung, liver, and brain) across all postmortem time points was assessed using the Shapiro–Wilk test. Multivariate normality assumptions were evaluated using Mardia’s skewness and kurtosis tests. Due to significant deviations from normality in raw ethyl chloride concentration values, a Box–Cox transformation was applied to stabilize variance and approximate a normal distribution. The transformation was applied to raw ethyl chloride concentrations measured in each matrix and time point to correct for heteroscedasticity prior to parametric statistical modeling. All ethyl chloride concentrations were normalized and reported as nanograms per 100 mg of tissue or per 0.1 mL of blood, consistent with established conventions in postmortem forensic toxicology. Following transformation, repeated measures analysis of variance (RM-ANOVA) and multivariate analysis of variance (MANOVA) were employed to assess the effects of time and matrix type on ethyl chloride concentrations. Pairwise comparisons were conducted using Pillai’s Trace and Bonferroni adjusted post hoc tests where appropriate. Statistical significance was defined as *p* < 0.01 for assumption testing and *p* < 0.05 for hypothesis testing.

## 3. Results

### 3.1. Method Validation Study Results

The matrix-matched method validation protocol yielded high analytical performance across four biologically relevant postmortem matrices: blood, lung, liver, and brain. Calibration curves established for each matrix over five concentration levels (0.2, 0.5, 1.0, 1.5, and 2.0 ng/µL) exhibited excellent linearity, with coefficients of determination (R^2^) ranging from 0.9947 (lung) to 0.9965 (liver), indicating robust and reproducible detector response across for diverse biological samples (Table 2). Peak area ratios of ethyl chloride to the internal standard (n-propanol) were consistent within and across matrices, confirming calibration stability and matrix compatibility.

The method demonstrated high sensitivity, with limits of detection (LOD) between 0.01 and 0.02 ng/µL and limits of quantification (LOQ) between 0.04 and 0.06 ng/µL, empirically determined via replicate (n = 10) analysis of low-level fortified samples. Intraday and interday precision, assessed at three QC levels (0.2, 0.8, and 2.0 ng/µL), yielded relative standard deviation (RSD%) values ranging from 3.9% to 5.1%, and accuracy (bias%) values within ±2.2% for all matrices, fully meeting the Eurochem acceptance criteria (≤±20%).

No matrix-induced retention shifts or co-eluting interferences were observed. Ethyl chloride and n-propanol retention times remained stable across matrices, averaging 2.59 ± 0.01 min and 4.15 ± 0.02 min, respectively. Selectivity was confirmed by complete baseline separation of ethyl chloride in the presence of volatile interferents (ethanol, methanol, acetone, and isopropanol), with no observable cross-reactivity or suppression. 

Absolute recovery values ranged from 90% (brain) to 104% (liver), with minimal inter-matrix variability, demonstrating reliable analyte extraction and consistent HS-GC-FID performance. No analyte carryover was detected in post high-concentration sample injections, further confirming method cleanliness.

Collectively, these findings validate the method’s robustness, reproducibility, and applicability for detecting trace levels of ethyl chloride in complex biological matrices. The results establish a high-performance framework for forensic toxicological investigations of volatile substance exposure under postmortem conditions.

### 3.2. HS-GC Results

**Figure 2:**
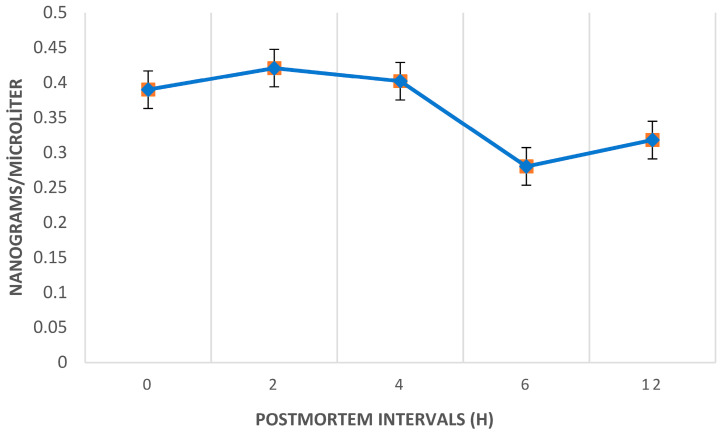
Mean Ethyl Chloride Concentration (ng/0.1 mL for blood, ng/100 mg for tissues) Across Five Postmortem Intervals (0, 2, 4, 6, and 12 h).

The average ethyl chloride levels in the postmortem period peaked at the 2nd hour across all samples. Figure 2 illustrates the temporal trend of postmortem ethyl chloride levels, highlighting a significant decrease between the 4th and 6th hours, with levels dropping substantially below the initial levels by the 6th and 12th hours.

**Figure 3:**
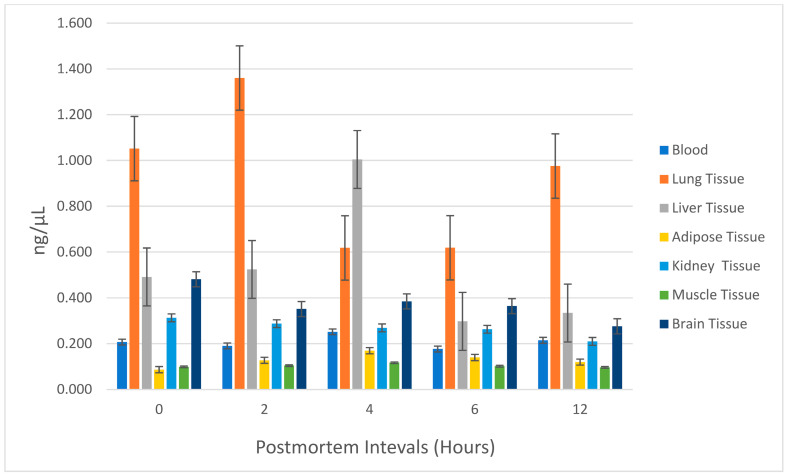
Comparison of Ethyl Chloride Concentrations Among Different Tissues Regardless of Time Points.

**Figure 4:**
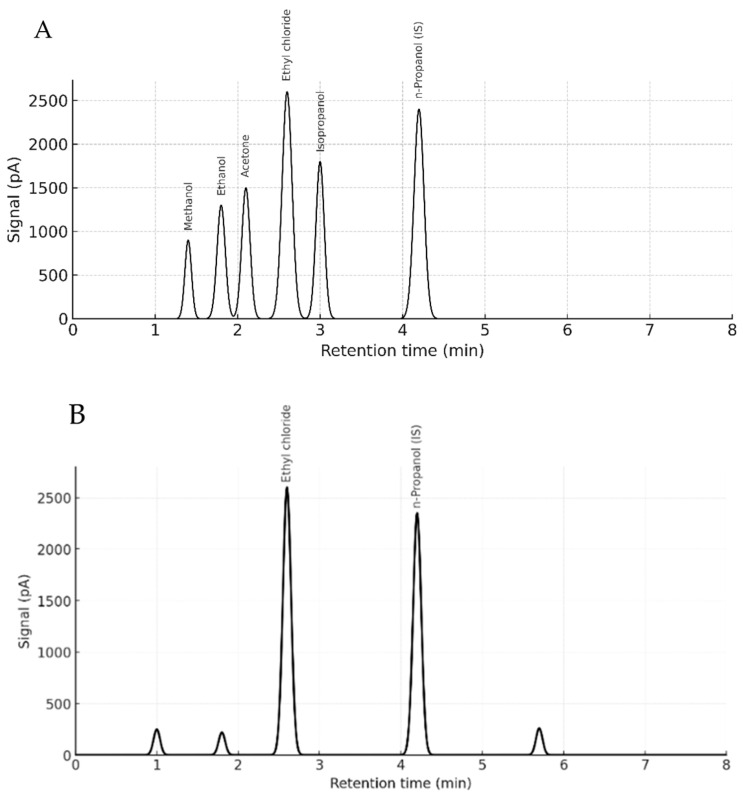
(**A**,**B**). Representative chromatograms of ethyl chloride and volatile interferents in standards and biological matrices. (**A**) Mixed standard chromatogram containing ethyl chloride, ethanol, methanol, acetone, and isopropanol (with n-propanol as the internal standard), demonstrating complete baseline separation of all volatile compounds. The ethyl chloride and n-propanol peaks elute at approximately 2.6 and 4.2 min, respectively. (**B**) Representative biological sample chromatogram (blood, 2 h postmortem) showing the ethyl chloride peak in a real postmortem matrix together with the internal standard (n-propanol).

The lung exhibited the highest ethyl chloride concentration among all tissues sampled. At 0, 2, 6, and 12 h postmortem, the lung tissue consistently showed the highest ethyl chloride levels, followed by liver and brain tissues. However, at the 4th hour postmortem, liver tissue displayed the highest ethyl chloride concentration. Ethyl chloride levels generally decreased between 4 and 6 h postmortem in all tissues except for the lung. Postmortem ethyl chloride levels became irregular after the 6th hour. Between 6 and 12 h postmortem, ethyl chloride levels increased in blood, lung, and liver tissues, while a decrease was observed in adipose, kidney, muscle, and brain tissues. The variations in ethyl chloride levels across different tissues and time points are further illustrated in Figure 3, providing a detailed visual representation of the observed trends and suggesting organ-specific differences in ethyl chloride accumulation and postmortem redistribution, particularly in lung, liver, and brain. No statistically significant relationship was found between postmortem ethyl chloride levels and time (*p* = 0.629), with observed differences attributed to tissue origin. Representative chromatograms illustrating retention time stability and complete baseline separation of ethyl chloride and volatile interferents in both standards and biological matrices are presented in Figure 4A,B.

In comparison, the levels of ethyl chloride in the samples regardless of time revealed that lung tissue exhibited the highest concentrations among all postmortem samples, with statistically significant differences compared to blood, adipose, kidney, and muscle tissues (*p* < 0.05). Liver and brain tissues also showed higher levels than adipose and muscle tissues, further highlighting their relevance for toxicological analyses. Adipose and muscle tissues consistently demonstrated the lowest concentrations, limiting their applicability in forensic investigations.

Although no statistically significant relationship was found between ethyl chloride levels in the samples and time points overall, the power of the test used to determine the detectable differences was low (power = 0.202). To improve analytical power, a multivariate approach was employed while ensuring assumption compliance. In multivariate analysis, a statistically significant difference was observed in the ethyl chloride levels in lung tissue between the 2nd and 4th hours postmortem, as determined by Pillai’s Trace test (*p* = 0.034, Observed Power = 0.778, Partial Eta Squared = 0.983).

## 4. Discussion

Toxicological evaluation in cases of death due to inhalant abuse of volatile substances presents significant challenges. Prompt postmortem analysis is crucial for detecting these substances and determining the cause of death [27,28]. HS-GC provides exceptional specificity, sensitivity, and accuracy for detecting and analyzing a range of volatile substances, including ethyl chloride, toluene, butane, propane, methane, and ethanol [27,29,30,31]. While HS-GC has been applied to various volatile substances, methods specifically for detecting ethyl chloride are limited in the literature [31,32,33]. The validated method introduced in this study represents a significant advancement, offering a reliable approach for analyzing ethyl chloride in postmortem biological samples. This methodology enhances the ability to detect and quantify ethyl chloride, thus providing a valuable tool for forensic toxicology, especially in cases involving suspected ethyl chloride exposure. By establishing a robust analytical framework, this study contributes to the field’s understanding of ethyl chloride’s behavior in postmortem conditions and its implications for cause-of-death investigations. One of the primary challenges in applying the validated method to real forensic cases lies in the rapid postmortem redistribution and degradation of ethyl chloride, which necessitates timely sample collection and analysis to ensure accuracy. On the other hand, despite the absence of validated methods using human tissue, this study emphasizes the importance of future research and broader collaborations to enhance the applicability of the method. Expanding research into further validated methods with similar volatile substances may also enhance the method’s generalizability and applicability in forensic toxicology.

Although GC-MS is generally preferred for compound identification due to its mass spectral resolution, this study highlights HS-GC-FID’s clear advantages for volatile compound screening, especially under matrix-matched and controlled conditions. Specifically, HS-GC-FID provides higher injection throughput, simpler maintenance, faster run times, and lower operational costs, making it particularly valuable for routine forensic toxicology laboratories where mass spectrometric platforms are not always available. Moreover, in the context of ethyl chloride, an analyte with high volatility and limited fragment ion information, FID detection offers robust peak integration and signal stability, especially when matrix-matched calibration and blank biological controls are used. In this study, method validation was performed using blank rat tissues (blood, lung, liver, and brain), with no observable endogenous or exogenous volatile interferences. Notably, commonly encountered volatile substances such as ethanol, methanol, acetone, and isopropanol were chromatographically separated without co-elution. These findings reinforce the suitability of HS-GC-FID as a cost-effective, time efficient, and analytically robust alternative for targeted detection of ethyl chloride, particularly in forensic scenarios requiring rapid screening and quantification. This framework provides a systematically validated, matrix-matched HS-GC-FID approach for postmortem ethyl chloride under controlled exposure conditions, offering translational guidance for routine forensic workflows.

In previous case reports of death due to abuse of death due to ethyl chloride abuse via spray cans, autopsies were typically conducted approximately 12 h postmortem, with no detailed assessment of the autopsy samples provided [13,14,34]. In the present study, all samples were collected throughout the first 12 h postmortem, acknowledging potential difficulties in detecting volatile substances due to decomposition and environmental factors in the postmortem process. Our findings indicate that the highest concentration of ethyl chloride was detected at the 2nd hour postmortem. While there was no statistically significant difference between ethyl chloride levels in postmortem samples over time, a significant decrease in ethyl chloride levels was observed between the 4th and 6th hours postmortem, with levels dropping below those measured at the 0th hour by the 6th hour. This suggests that, for efficient postmortem investigations, autopsies should ideally be conducted within the first 6 h postmortem to maximize the likelihood of detecting ethyl chloride.

Ethyl chloride tends to accumulate in air-filled lung tissue, which is the first organ that inhaled-volatiles contact in the body. It also tends to accumulate in brain cells due to their lipophilic properties and the liver tissue where it is metabolized [35,36,37]. Masakazu and Mitsukini reported that lung and brain tissues showed the highest concentrations of ethyl chloride in a postmortem case [34]. Lehman and Flury showed that ethyl chloride accumulated in the brain at levels twice that of blood, with significantly high concentrations in the brain and medulla oblongata [38]. Todd et al. reported a temporary mild hepatic dysfunction in a woman who had used inhaled ethyl chloride for four months [37]. This study revealed that ethyl chloride accumulates at the highest level in the lung tissue compared to other samples. Taken together with the time–concentration profiles shown in Figure 3, these findings indicate a clear organ specificity: lung acts as the primary depot for inhaled ethyl chloride, followed by liver and brain, whereas adipose, kidney, and muscle show only low and often irregular levels. This pattern is consistent with the high perfusion and air content of the lung, the metabolic role of the liver, and the marked lipophilicity of ethyl chloride, which favors rapid CNS uptake. Moreover, when postmortem samples were compared separately at each time point, the level of ethyl chloride in the lung tissue was found to be the highest among all other samples except for liver tissue only at the postmortem 4th hour. In the comparison of postmortem tissue ethyl chloride levels regardless of time, lung tissue showed the most significant results. These results are consistent with the available data in the literature. The statistical outcomes of this study illuminate the pivotal role of lung tissue as a dependable biological sample for postmortem detection of ethyl chloride. The inherent properties of lung tissue, notably its air-filled architecture and capacity for retaining volatile compounds, render it uniquely suited for toxicological investigations involving volatile substances. The observed fluctuations in ethyl chloride concentrations between the 2nd and 4th postmortem hours accentuate the critical importance of early sampling intervals to maximize the reliability of toxicological assessments. Moreover, a comparative contextualization with existing studies on other volatile compounds further underscores the broader applicability of these insights, enhancing the methodological framework for forensic investigations into inhalant related fatalities. Although the findings of this study are derived from a controlled animal model, species-specific metabolic differences should be considered when interpreting the detectability and distribution of ethyl chloride.

Between 2000 and 2021, a comprehensive study conducted in Australia examined 164 deaths associated with volatile solvent misuse. Among these, butane was identified as the most frequently implicated substance, accounting for 42 cases, followed by toluene with 32 cases, and propane with 27 cases. Furthermore, autopsy findings were reviewed for 104 cases, revealing that pulmonary edema was the most prevalent diagnosis, observed in 70.2% of cases. This study underscores the critical role of lung tissue in volatile substance-related fatalities, highlighting its significance both macroscopically and toxicologically as a valuable sample for postmortem analysis [39].

Although studies on inhalant toxicity in the literature are more commonly focused on chronic exposure, there are also investigations addressing acute inhaler toxicity [40]. Volatile substances such as butane and toluene are frequently encountered in fatalities associated with solvent misuse [41]. Comparative studies on substances such as butane and toluene have demonstrated similar challenges in postmortem detection due to their volatile nature and rapid redistribution. For instance, a systematic review of butane-related deaths reported pulmonary edema in 51% of cases, underscoring the lung as a critical organ for volatile substance analysis [42]. This underscores the critical need for standardized protocols in detecting and interpreting volatile substances, with lung tissue highlighted as a key tissue sample in postmortem forensic analyses. The limited sample size may have influenced the detection of statistically significant differences in ethyl chloride distribution across tissues and time intervals. However, multivariate analysis revealed significant differences in lung tissue ethyl chloride levels between the 2nd and 4th hours postmortem, underlining the lung as the most reliable organ for autopsy sampling in cases of ethyl chloride related fatalities.

The second most detectable sample for postmortem ethyl chloride was the liver, where the highest concentration of ethyl chloride was determined at postmortem 4th hour. This finding could be attributed to the extensive metabolism of ethyl chloride in the liver. Similarly, our study identified brain tissue as the third most accumulating tissue for ethyl chloride, following the lungs and liver. The quantities of ethyl chloride detected in the brain tissue varied over the postmortem period, but between 0 and 6 h postmortem, the levels in the brain were approximately twice those detected in the blood. Comparative studies on other volatile substances, such as butane and toluene, support our findings regarding the role of the liver in postmortem toxicological analyses. In a study examining butane toxicity, postmortem analysis highlighted detectable butane levels in the liver, underscoring its role in metabolizing volatile compounds [43]. Similarly, research on toluene demonstrated significant accumulation in the liver and brain postmortem, further validating their importance in the metabolism and storage of volatile substances [23]. These findings are consistent with our study’s results, emphasizing the liver and brain as crucial biological samples in postmortem forensic investigations of volatile substances. Although ethyl chloride is known to undergo biotransformation, especially in the liver, the present study was designed to quantify only the parent compound and did not include targeted analysis of its metabolites. Consequently, the organ distribution of ethyl chloride metabolites could not be assessed here. Future studies combining HS-GC-based quantification of the parent compound with mass-spectrometric identification of metabolites in liver, kidney, and brain would be valuable to further elucidate the full toxicokinetic profile of inhaled ethyl chloride.

It was reported that the highest concentration of ethyl chloride in animals was observed in the perirenal adipose tissue [44]. In our study, perirenal adipose tissue was one of the three solid tissues along with kidney and muscle in which ethyl chloride was detected at the lowest levels in postmortem period. While lipophilic substances are known to accumulate in adipose tissue, it is not commonly used in routine acute toxicological analyses [45]. The literature reports the highest ethyl chloride concentration in animals was detected in the perirenal adipose tissue; however, this pertains to chronic exposure [44]. In our study, the adipose tissue was perirenal adipose tissue. The postmortem levels of ethyl chloride in adipose and muscle tissues increased between 0 and 4 h, followed by a decrease between 4 and 6 h. Additionally, the postmortem levels of ethyl chloride in the blood were found to be higher than those in adipose tissue at all time points. Since our study aimed to evaluate ethyl chloride levels in the acute period, we believe the lower detection levels in adipose tissue are consistent with this focus. Comparatively, studies on butane and toluene have demonstrated similar findings regarding postmortem blood concentrations. A systematic review analyzing deaths related to butane inhalation highlighted blood as an important biological sample for volatile substance analysis [42]. Similarly, research on toluene toxicity identified high concentrations in postmortem blood, further emphasizing its importance in acute exposure cases [41]. These findings align with our observation of higher ethyl chloride levels in blood compared to adipose tissue, underscoring blood’s value as a key biological sample in postmortem toxicological investigations.

Ethyl chloride is rapidly absorbed from the lungs due to its chlorinated hydrocarbon structure. With its high lipophilic property, it allows rapid absorption in the brain and then redistribution to the body. After absorption into the brain, central nervous system depression occurs rapidly, and at high concentrations, may result in sudden death. Due to the extremely volatile nature of ethyl chloride, the literature regarding postmortem redistribution of ethyl chloride is very limited [36]. Similarly, Kimura et al. investigated postmortem diffusion of thinner components, such as toluene, ethyl acetate, and isobutanol, and reported no significant changes in toluene and isobutanol concentrations within the thigh muscle over 48 h, despite gradual diffusion of these components into other tissues [46]. This may be associated with the sudden death of rats before they were able to fully achieve postmortem redistribution of ethyl chloride. In addition, a sudden increase in the concentration of ethyl chloride in the lung tissue until the 2nd hour, and the increase again between 6 and 12 h after the decrease between 2 and 6 h, may be associated with postmortem diffusion and redistribution.

## 5. Limitations

This study aimed to develop both a validated analytical method and a controlled animal model to assess postmortem ethyl chloride concentrations across diverse autopsy samples. Method validation was primarily performed using matrix-matched calibration curves derived from blank (non-exposed) rat tissues namely, blood, lung, liver, and brain to address potential matrix effects caused by endogenous volatiles and nonvolatile biological compounds. These tissues were directly spiked with ethyl chloride under strict cold-chain conditions and analyzed using HS-GC-FID, thereby ensuring minimal sample preparation and reducing volatilization-related analyte loss. Calibration results demonstrated high linearity, precision, and reproducibility, with no significant matrix-related interference observed. However, matrix-matched validation was not extended to adipose, muscle, or kidney tissues due to the limited availability of sufficient high-purity blank tissues and the heightened risk of baseline volatility interference in these matrices. This may be considered a methodological limitation. Future studies could enhance forensic applicability by incorporating matrix validation for these additional tissue types.

A further limitation lies in the exposure protocol design, which employed a fixed ethyl chloride concentration (86,000 ppm) over a short, predefined time interval. While this protocol ensured experimental reproducibility and alignment with established LC_50_-based toxicological models, it did not simulate a range of exposure dynamics that might occur in real-life scenarios. Designing dose–response or variable-duration exposure protocols would offer a more comprehensive toxicity profile, but would require a larger animal cohort and additional ethical considerations.

Additionally, the study’s sample size may have limited the statistical power to detect subtle differences in ethyl chloride distribution across time points and tissue types. While multivariate analyses revealed significant temporal fluctuations, particularly in lung tissue, larger sample cohorts would bolster both the statistical robustness and generalizability of these findings. Despite these limitations, this study introduces a rigorously validated analytical approach and offers a reproducible experimental framework, providing foundational evidence to inform future research into the forensic toxicology of volatile substances, particularly in postmortem investigations involving ethyl chloride.

An additional practical limitation concerns the recommendation to perform autopsies and collect postmortem samples within the first 6 h after death to optimize ethyl chloride detectability. In many jurisdictions, current legal and logistical frameworks make such early autopsies difficult or impossible to implement systematically. In this context, our findings suggest that, if early full autopsy is not feasible, collecting femoral or cardiac blood at the time of body removal and during evisceration at autopsy may offer a pragmatic compromise to capture ethyl chloride while still respecting local legal constraints.

## 6. Conclusions

This study establishes the first experimental framework for systematically evaluating the postmortem distribution and temporal dynamics of ethyl chloride using a rigorously validated matrix-matched HS-GC-FID method. It addresses a critical gap in forensic toxicology by introducing both methodological innovation and empirical evidence for the detection of volatile anesthetics in postmortem investigations. Among all matrices, lung tissue consistently exhibited the highest ethyl chloride concentrations, with peak levels at 2 h postmortem and a statistically significant decline between the 4th and 6th hours. These findings underscore the necessity of early sampling, preferably within 6 h after death, to preserve toxicological integrity. The lung, liver, and brain were identified as the most diagnostically informative matrices, owing to their lipid-rich composition and functional relevance. In contrast, adipose and muscle tissues demonstrated low and inconsistent analyte retention, limiting their utility in forensic contexts. The HS-GC-FID platform, characterized by high sensitivity, specificity, and reproducibility, offers a cost-effective and operationally feasible alternative to mass spectrometry-based approaches in volatile substance analysis. Importantly, the controlled rat model enabled precise characterization of ethyl chloride pharmacokinetics under standardized conditions, thereby enhancing the translational validity of the findings. Although species-specific differences must be considered, this study provides foundational data supporting the prioritization of lung tissue and early postmortem intervals in forensic protocols involving volatile substance misuse. It advances the methodological rigor of forensic toxicology and offers a benchmark for future research targeting the detection and interpretation of highly volatile compounds in medico-legal settings.

## Figures and Tables

**Table 1:** Analytical Conditions for the HS-GC Method Used in the Detection of Ethyl Chloride in Postmortem Samples.

Components	Parameters	Requirement
**Autosampler**	Vial equilibrium time	20 min
	Loop fill time	0.15 min
	Loop equilibrium time	0.05 min
	Injection time	0.50 min
	GC Cycle Time	13.5 min
**Inlets**	Heater	90 °C
	Pressure	9.2324 psi
	Total flow	15 mL/min
	Septum purge flow	3 mL/min
	Split Ratio	5:1, 10 mL/min
**Column**	Dual Column DB-Alc1 DB-Alc2	30 m × 320 μm × 1.8 μm 30 m × 320 μm × 1.2 μm
	Flow	2.0305 mL/min
	Average velocity	32.97 cm/sec
	Oven temperature program	2 min-35 °C 25 °C/min 8.5 min-220 °C
	Equilibrium time	3 min
**Detector**	Front detector FID	260 °C
	Back detector FID	260 °C
	H_2_ flow	40 mL/min
	Airflow	450 mL/min
	Makeup flow (He)	48 mL/min

**Table 2:** Matrix-Matched Method Validation Parameters for Ethyl Chloride Quantification in Postmortem Blood, Lung, Liver, and Brain Tissues.

Matrix	R^2^	LOD (ng/µL)	LOQ (ng/µL)	Intra-Day RSD%	Inter-Day RSD%	Bias%	Recovery %
**Blood**	0.9961	0.02	0.06	4.8	5.1	+1.4	92
**Lung**	0.9947	0.02	0.05	5.1	4.3	−2.2	94
**Liver**	0.9965	0.01	0.04	3.9	4.6	+1.1	104
**Brain**	0.9952	0.01	0.05	4.5	5.0	+0.7	90

## Data Availability

The data supporting the findings of this study are not publicly available due to institutional and ethical restrictions related to animal experimental records.
